# Progress in Mycotoxins Affecting Intestinal Mucosal Barrier Function

**DOI:** 10.3390/ijms20112777

**Published:** 2019-06-06

**Authors:** Zhihua Ren, Chaoyue Guo, Shumin Yu, Ling Zhu, Ya Wang, Hui Hu, Junliang Deng

**Affiliations:** 1 Key Laboratory of Animal Disease and Human Health of Sichuan Province, College of Veterinary Medicine, Sichuan Agricultural University, Chengdu 611130, China; zhihua_ren@126.com (Z.R.); Guochaoyue_1997@126.com (C.G.); yayushumin@163.com (S.Y.); abtczl72@126.com (L.Z.); wangyayang@126.com (Y.W.); 2 The College of Animal Science and Veterinary Medicine, Henan Agricultural University, Zhengzhou 450002, China

**Keywords:** mycotoxins, intestinal mucosa, intestinal barrier

## Abstract

Mycotoxins, which are widely found in feed ingredients and human food, can exert harmful effects on animals and pose a serious threat to human health. As the first barrier against external pollutants, the intestinal mucosa is protected by a mechanical barrier, chemical barrier, immune barrier, and biological barrier. Firstly, mycotoxins can disrupt the mechanical barrier function of the intestinal mucosa, by destroying the morphology and tissue integrity of the intestinal epithelium. Secondly, mycotoxins can cause changes in the composition of mucin monosaccharides and the expression of intestinal mucin, which in turn affects mucin function. Thirdly, mycotoxins can cause damage to the intestinal mucosal immune barrier function. Finally, the microbiotas of animals closely interact with ingested mycotoxins. Based on existing research, this article reviews the effects of mycotoxins on the intestinal mucosal barrier and its mechanisms.

## 1. Introduction

Mycotoxins are secondary metabolites produced by filamentous fungi or molds during grain growth, harvesting, storage and processing, including deoxynivalenol (DON), aflatoxin B1 (AFB1), zearalenone (ZEA), ochratoxin A (OTA), T-2 toxin and fumonisin B (FBs) [1]. These metabolites are common natural biological pollutants found in human and animal diets. They widely exist in feed materials and livestock and poultry feeds worldwide and are prone to acute and chronic poisoning of humans and animals. Although people have already realized the harm of mycotoxins to livestock and poultry and reduced the chances of pollution as much as possible in production practice, the pollution of mycotoxin is still unavoidable, and the effect of existing detoxification technology is unsatisfactory. In 2018, the mycotoxin detection report of raw materials and feed of BIOMIN Feed Additives (Shanghai) Co., Ltd. (BIOMIN) showed that trichothecene toxin B group (represented by vomiting toxin) and fumonisin were the most common contamination in maize, with detection rates of 91% and 93%, respectively. Zearalenone and aflatoxin were also highly contaminated toxins [2], which posed a serious threat to the health of animals and human beings [3,4,5,6,7].

The Gastrointestinal (GI) tract is an organ within humans and other animals, which is responsible for food ingestion, digestion, energy and nutrient absorption, immune response, as well as elimination of waste products (feces) [8]. The digestive tract is the first organ to come into contact with food, and mycotoxins are mainly absorbed through the intestine. Because the digestive tract is the first organ to be exposed to mycotoxins, it is also the first to suffer toxic damage [9,10]. As the first biological barrier of the intestine, the intestinal mucosa also functions as a mechanical, chemical, and immunological barrier. The epithelium layer at the innermost part of the mucosa is of vital importance for intestinal barrier function. It acts as a barrier to the entry of harmful agents such as pathogens, toxins, and foreign antigens [11]. The Adherens Junctions (AJs) and desmosomes are responsible for the mechanical linkage of adjacent cells. Whereas, the Tight Junctions (TJs) control the intercellular space and regulate selective paracellular ionic solute transport. Above the epithelium lies a complex microflora which is recognized as gut microbiota [12]. The gut microbiota allows the host to metabolize a vast range of dietary substrates. For example, the metabolism of carbohydrate is a major catalytic function of the microbiota. The gut microbiota assists in the fermentation of complex polysaccharides that escaped proximal digestion [13]. Apart from that, the gut microbiota protects the host against infections via several mechanisms. When the intestinal mucosal barrier is dysfunctional, it not only causes intestinal inflammation, but also greatly increases the chance of exposure to exogenous chemicals and pathogens [14].

With the development of research, people are increasingly aware of the harmful effect of mycotoxins on the gastrointestinal tract. The most in-depth study is to destroy the integrity of the intestinal epithelium, which can be proven by changing the morphology and structure of intestinal epithelium, reducing transepithelial resistance (TEER), and reducing the expression of tight junction protein. For the other two important components of the intestinal epithelium: Mucus and microflora, mycotoxins can reduce the expression of mucin and change the abundance and structure of intestinal microflora. This article reviewed the effects of mycotoxins on the intestinal mucosal barrier and its mechanism of action, providing a theoretical basis for further research in this field.

## 2. Effect of Mycotoxins on the Mechanical Barrier Function of the Intestinal Mucosa

The intestinal mucosal mechanical barrier is mainly composed of intestinal epithelial cells and tight junction proteins that exist between them. It is not only key to protecting the intestinal tract against invasion of the intestinal mucosa by pathogens or harmful substances in the external environment, but also forms the structural basis for maintaining the selective permeability of the intestinal epithelium and its barrier function. The intestinal epithelium is the largest epithelium on the surface of human mucosa [15], and its cells have a rapid value-adding and regenerative ability to maintain intestinal mucosal mechanical barrier function [16]. Studies have shown that mycotoxins can disrupt the mechanical barrier function of the intestinal mucosa, by destroying the morphology and tissue integrity of the intestinal epithelium. The in vitro and in vivo studies of the effects of different mycotoxins on the intestinal mucosal mechanical barriers of different species used in the literature cited herein are shown in Table 1. The toxic effects of mycotoxins on the morphological structure of the intestinal mucosa are shown in Table 2.

Tight junctions play an important role in the attachment of epithelial cells, maintaining epithelial cell structure, and thereby the biological function of epithelial cells. Mycotoxins can exert toxicity by destroying the integrity of the intestinal mucosal mechanical barrier. Wu et al. [30] fed healthy broilers aged 1 day with 10mg/kg DON and found that the mRNA expression of cloudin-1 and occludin (*p* < 0.05),one of the genes in ileum which is involved in nutrient transport *rBAT* and the glucose transporter protein 1 (GLUT1) mRNA in jejunum of 21-day-old broiler chickens decreased significantly (*p* < 0.05). The expression of duodenal PEPT1 and ileum rBAT in 42-day-old broilers decreased significantly. Bracarense and colleagues [24] fed 5-week-old piglets a low dose (3 mg/kg) of DON or a low dose (6 mg/kg) of FB1 for 5 weeks, and the results showed that expression of the tight junction protein occludin in the small intestine was significantly reduced. However, experiments such as those conducted by Martin Lessard [35] has suggested a deeper indication that the genes of claudin, occludin and vimentin in the ileum of pigs fed DON-containing feed are down-regulated. Diesing and coworkers [36] showed that after 2000 ng/mL DON was applied to the polarized epithelial cells of porcine small intestinal origin (IPEC-1 and IPEC-J2), the expression of tight junction protein ZO-1 decreased, and the intestinal mechanical barrier integrity was destroyed. In vitro Caco-2 cell test showed that the mycotoxins such as AFB1, FB1, OTA and T2 reduced the transepithelial electrical resistance while the expression levels of claudin-3, claudin-4 and occludin mRNA were also reduced [34].

**Figure 1:**
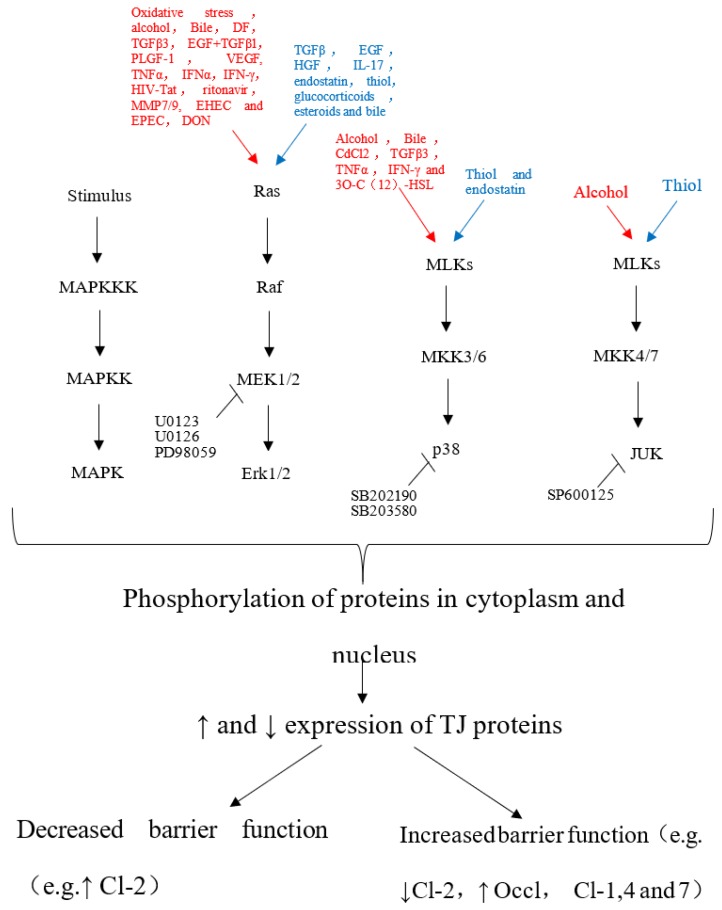
The agent that activates the cascade in the MAPK pathway, which can lead to TJs opening or assembly. The uppermost portion of the figure indicates the activators of the MAPK pathway that leads to TJs disassembly (red) or that favor TJs tightening (blue). The hierarchical organization of MAP signaling cascades into three-tiered modules of MAPKKK, MAPKK and MAPK is shown [38].

Pinton and coworkers [37] found that DON acted on IPEC-1 cells by activating extracellular regulated protein kinases (ERK) in the mitogen-activated protein kinase (MAPK) signaling pathway. Furthermore, inhibition of the synthesis of the tight junction protein claudin-4 destroyed the integrity of the intestinal mucosal mechanical barrier. The toxicity of DON differs depending on its structure, for example, 15-acetyl deoxynivalenol (15-ADON) is more toxic to the intestines than DON and 3-acetyl deoxynivalenol (3-ADON) because it activates MAPK [19]. The signal network diagram of the MAPK pathway regulating tight junction proteins is shown in Figure 1 [38].

The main mechanisms of mycotoxin damage to intestinal cells are oxidative damage and DNA damage. Bensassi and colleagues [39] monitored the effects of DON on (i) viability of human colon cancer cells, (ii) heat shock protein expression as a parameter for protective and adaptive responses, (iii) oxidative damage, and (iv) the cell death signal conductive pathway. The results not only clearly indicated that DON inhibited cell proliferation, but also that DON induced DNA fragmentation followed by p53 and caspase-3 activation. The results also indicated that oxidative damage was not the major cause of DON toxicity, which induced direct DNA damage and could be considered a genotoxic agent that induced cell death through an apoptotic process. Taranu and colleagues [40] performed transcriptome analysis of IPEC-1 in pig intestinal epithelial cells treated with 10 μmol/L Zearalenone (ZEA). At this concentration, the survival rate of IPEC-1 cells was not affected, and ZEA could regulate glutathione peroxide. Expression of the enzyme encoded by the *GPx* gene (*GPx6, GPx2, GPx1*) promotes the production of reactive oxygen species. Under conditions that do not affect cell viability, mycotoxins also cause oxidative damage to cells by destroying DNA. Oxidative damage is the end point, but changes to the DNA are the intrinsic result of mycotoxin activity. Damage to cellular DNA by mycotoxins can be inhibited by the addition of antioxidants. After Abid-Essefi and coworkers [41] applied ZEA to undifferentiated Caco-2 cells, agarose gel electrophoresis revealed that ZEA caused DNA fragmentation in a dose-dependent manner, inhibited the formation of DNA adducts, and producing a ladder-like DNA pattern; however, the addition of vitamin E, shortened the cell cycle, reduced the production of DNA fragments, and repaired the DNA damage. Vitamin E uses its antioxidant activity to repair DNA damage in cells, which indicates that oxides are closely linked to DNA damage.

## 3. Effect of Mycotoxins on Intestinal Chemical Barrier Function

The chemical barrier in the intestines consists of gastric acid, bile, various digestive enzymes, lysozymes, mucopolysaccharides, and other chemical substances secreted by the gastrointestinal tract and bacteriostatic substances produced by intestinal parasites. The secretion of highly glycosylated mucin into the intestinal lumen by goblet cells creates a first line of defense against microbial invasion. These mucins play an important role in the entry of foreign pollutants into the deep tissue of the intestinal mucosal barrier [10,15]. The in vitro and in vivo studies on the effects of different mycotoxins on the intestinal mucosal chemical barriers of different species used in the literature cited in this paper are shown in Table 3.

Studies have shown that mycotoxins can cause changes in the composition of mucin monosaccharides in chicken intestines, which in turn affects mucin function. Antonissen and coworkers [42] found that mycotoxins FB and DON changed the proportion of monosaccharides in the duodenal mucin in broilers, affecting the function of the mucus layer of the duodenum. The results of Applegate and colleagues [45] showed that low concentrations of mycotoxins also caused changes in the composition of intestinal mucosal monosaccharides in laying hens, and these changes may have helped to enhance intestinal resistance to mycotoxins.

In addition, studies have shown that mycotoxins (even at low concentrations) may affect mucin mRNA expression levels and protein expression levels, leading to intestinal mucus damage and intestinal inflammation. Wan and coworkers [43] found that the mixed toxin of DON and ZEA increased the mRNA expression level of goblet-specific protein MUC2. Furthermore, the effects of mycotoxins DON, ZEA, NIV, and FB1, alone or in combination, on the ratio of intestinal epithelial mucin indicated that mycotoxins could significantly alter the mRNA and protein expression levels of MUC5AC and MUC5B. The degree of change at the protein level was similar to or less than that at the transcriptional level [9]. This suggested that at least some of the post-transcriptional or post-translational regulatory mechanisms were closely related to the mycotoxin-induced molecular synthesis and secretion of mucin. In addition, Pinton and colleagues [44] used human goblet cells (HT29-16E) and porcine small intestine explants as experimental subjects and found that MUC1, MUC2, and MUC3 mRNA and protein expression were observed after exposure to the mycotoxin DON for 48 h, and effects on the intestinal mucosal barrier were observed.

Mammalian intestinal tracts enhance their barrier function through intestinal epithelial cells (IECs) secreting antimicrobial peptides. Paneth cells are particularly well-suited for the secretion of many antimicrobial peptides, including defensins in the small intestine crypt (crypt proteins in mice), cathepsins, and lysozyme [15]. Studies have shown that mycotoxins cause upregulation of the mRNA expression of porcine defensins 1 and 2 (pBD-1 and pBD-2) (*p* < 0.05) [46]. From this, it can be concluded that mycotoxins cause upregulation of intestinal antimicrobial peptide secretion. An in vitro study by Han and coworkers [47] found that porcine beta-defensin 2 (pBD2) increased mucin mRNA expression in Caco-2 cells. Therefore, we inferred that when the intestinal tract was damaged by mycotoxins, the expression of mucin mRNA increased by upregulating the secretion of antimicrobial peptides, thereby enhancing the chemical barrier function of the intestinal mucosa. In addition, a large number of studies have confirmed that the addition of antimicrobial peptides to feed can antagonize the toxicity of mycotoxins. The experimental results of Xiao and colleagues [48] showed that the addition of compound antimicrobial peptide (CAP) to feed could improve intestinal morphology, and promote intestinal epithelial cell proliferation and protein synthesis, indicating that CAP could repair DON-induced intestinal damage. The results of Hongbo’s work [49] also showed that the antimicrobial peptide CWA could improve the self-repair ability of damaged intestinal epithelial cells and the barrier function of the intestinal mucosa.

## 4. Effect of Mycotoxins on Intestinal Mucosal Immune Barrier Function

The immune barrier consists primarily of a population of cells of the intestinal immune system, including cells derived from intestinal-associated lymphoid tissues and disseminated immune cells. The former mainly refers to the collecting lymphoid nodules distributed in the intestine, which are the induction and activation site of the immune response; the latter is the effector site of intestinal mucosal immunity. Secretory IgA is the main humoral immune component and effector molecule on the gastrointestinal and mucosal surfaces and is the first line of defense against adhesion and colonization of the intestinal mucosa. Studies have shown that mycotoxins can damage the humoral and cellular immunity of the intestinal mucosa. The experimental dose and damage effects are shown in Table 4 and Table 5, respectively.

Mycotoxins increase the secretion of pro-inflammatory factors, leading to increased permeability of the intestines, making it easier for certain factors in the gut to enter the bloodstream through the intestine [59]. Injury effect of mycotoxins on intestinal mucosal immune barrier may be affected by factors such as the type of mycotoxin, differences in the dose of toxins and the time of exposure, and differences between animal species [61,62,63].

## 5. Effect of Mycotoxins on Intestinal Mucosal Biological Barrier Function

The biological barrier in the intestines is formed by the resident intestinal flora that co-inhabits the intestinal lumen or colonizes the surface of the intestinal mucosa. Under normal conditions, a large number of anaerobic bacteria grow on the surface of the intestinal mucosa, such as *Bifidobacterium*, which can bind closely to the intestinal epithelium through adhesion, forming a membrane barrier that can resist and repel the invasion of exogenous pathogens. The production of short-chain fatty acids, acetic acid, lactic acid, and other nutrients by intestinal mucosal cells, reduce the intestinal pH, activate the intestinal immune system, and play an important role in maintaining intestinal barrier function. If the balance of the intestinal microflora is disrupted, this may lead to a series of intestinal diseases such as diarrhea and enteritis. Interestingly, a few studies have shown that mycotoxins can alter the intestinal microbiota. In some studies, using methodology involving polymers, changes in the gut microbiota can be observed at the species levels. The study of different mycotoxins affecting the intestinal mucosal biological barrier of different species in the literature cited in this paper is shown in Table 6.

Many studies have shown that the microbiotas of animals closely interact with ingested mycotoxins. Microorganisms participate in the process of removing mycotoxins by metabolizing or binding the toxins. The results of Wang and colleagues [64] showed that AFB1 could induce changes in microbial community composition in a dose-dependent manner, thereby significantly changing the composition of the intestinal microflora (*p* < 0.05), and the content of some lactic acid bacteria was found to be significantly decreased (*p* < 0.05). Mingzhang Guo et al. [65] studied the effects of OTA on gut microbiota using metagenomics and culture-based methods. The results showed that OTA treatment reduced the diversity of gut microbiota and the relative abundance of *Lactobacilli* increased significantly. Piotrowska and colleagues [66] studied the effects of ZEA on the intestinal microbiota and found that the concentrations of *Clostridium perfringens*, *Enterobacteriaceae*, and *E. coli* significantly decreased (*p* < 005). Further experiments by Burel and coworkers [69] showed that the balance of the digestive tract microflora was temporarily, but significantly affected by short-term exposure to fumonisin mixed with FB1 and FB2 for 63 days. As for the effect of DON on intestinal microorganisms, studies have found that DON exposure reshapes the intestinal microbial structure and completely disrupts the abundance of several bacterial gates, families and genera, leading to imbalance of ecological balance [67,70]. Specifically speaking, Saint Cyr and coworkers [68] inoculated GF male rats with healthy human fecal flora to investigate the effects of DON on human intestinal microbes. The results showed that the concentration of *Bacteroides* increased significantly and the concentration of *Escherichia coli* decreased (*p* < 0.05) after 4 weeks of intragastric administration of DON. Lucke et al. [67] found that increasing DON levels linearly reduced high abundance of *Enterobacteriaceae* and *Escherichia coli/Shigella* (*p* < 0.05); Wu et al. [30] showed that the intestinal bacteria including *Proteus, Escherichia coli, CC-115* (*p* < 0.05), *Lactobacillus* and *Pasteurella pastoris* (*p* < 0.1) decreased after DON treatment. In particular, the genotoxicity of Escherichia coli-producing E. coli strains in the gut was regulated by DON in the diet, which was shown in a time- and dose-dependent manner in in vitro experiments. This provides a direction for studying the synergy between food contaminants and intestinal microbes in the development of intestinal cancer [70].

## 6. Summary

The intestinal tract is the first barrier in the body against the invasion of in vitro pollutants. High concentrations of contaminants such as mycotoxins can cause damage to the intestinal mucosa. If any of the four interconnected components of the intestinal barrier, that is, the mechanical, chemical, immune, or biological barrier, are damaged, this can lead to diseases such as intestinal inflammation and cancer. Therefore, it is necessary to control the content of mycotoxin in the diet, potentially by detoxifying feed during production. To date, more research has been reported on the mechanical, chemical, and immune barriers, than on the biological barrier, this may be due to technical difficulties in that the composition of the intestinal microbial community is greatly affected by various factors during the experiments. Factors affecting microbial composition and function include diet [71], environment, use of antibiotics [72], and genetic background [73]. In future research, we will seek a suitable experimental system in which to explore the interaction between mycotoxins and intestinal bio-barriers. An increased understanding of the mechanism of action will form a theoretical basis for the development of drugs to reduce intestinal injury caused by mycotoxins.

## Figures and Tables

**Table 1:** In vivo and in vitro studies on the effects of different mycotoxins on intestinal mucosal mechanical barriers in different species.

Mycotoxins	In Vitro/In Vivo	Species	Dose	Reference
DON	In vivo	broilers	10 mg/kg	[17]
		pigs	2.29 mg/kg	[18]
	In vitro	Caco-2	50, 100, 200 ng/mL	[19]
		the jejunal explant	10, 30 μM	[20]
AFB1	In vivo	broilers	0.6 mg/kg	[21]
	In vitro	Caco-2	1–100 μM	[22]
AFM1	In vitro	Caco-2	0.12 M, 12 M	[23]
OTA			1–100 μM	[22]
FB1			1–100 μM	[22]
T2			1–100 μM	[22]
FB1	In vivo	pigs	6 mg/kg	[24]

**Table 2:** Toxicity studies: The toxic effects of mycotoxins on the morphological structure of the intestines.

Mycotoxin	Species	Toxicity	References
DON	Broilers, rats, pigs	The villi of the duodenum and jejunum become shorter and thinner, flattened and fused, and apical necrosis	[17,18,25,26,27,28,29,30]
	Caco-2	(1) Decompose the Caco-2 cell monolayer; (2) Decrease the epithelial resistance; (3) Increase the permeability of the labeling molecule, such as Lucifer Yellow and FITC-labeled dextran; (4) Reduce the TEER.	[27]
	the jejunal explant	(1) The villi are flattened; (2) The adhesion and the lamina propria are weakened; (3) The epithelial cells are shed; (4) The scrotum is dilated.	[20]
AFB1	broilers	(1) Reduce jejunal villus density and intestinal absorption area; (2) Reduce jejunal villus height and villus height/crypt rate; (3) Reduce the goblet cell number; (4) Reduce the microvilli in intestinal absorption cells; (5) Mitochondrial vacuolization; (6) Mitochondrial ridges disappear; (7) Connection complex and terminal network disappear.	[21,31,32,33]
AFM1+OTA	Caco-2	Reduce transepithelial resistance	[23]
DON+ZEA	pigs	Reduce the number of goblet cells in the cecum and descending colon	[34]
DON+FB	pigs	(1) Villus atrophy and fusion; (2) Reduce the jejunum villus height and cell proliferation; (3) Decrease in goblet cells and lymphocytes.	[24]

**Table 3:** In vitro and in vivo studies on the effects of different mycotoxins on intestinal mucosal chemical barriers in different species.

Mycotoxins	In Vitro/In Vivo	Species	Dose	Reference
FBs	In vivo	broilers	25.4 mg (FB1+FB2)/kg feed	[42]
DON	In vivo	Broilers	4.6 mg/kg feed	[42]
		mouse	12 mg/kg	[43]
	In vitro	human goblet cells (HT29-16E)/porcine small intestine explants	1, 10, 100 μM	[44]
ZEA	In vivo	mouse	0.5 mg/kg	[43]

**Table 4:** In vitro and in vivo studies on the effects of different mycotoxins on intestinal mucosal immune barriers in different species.

Mycotoxins	In Vitro/In Vivo	Species	Dose	Reference
AFB1	In vivo	broilers	0.6 mg/kg	[50]
DON	In vivo	mouse	10 μg/kg BW	[51]
		pigs	3 mg/kg	[24]
			2.2–2.5 mg DON/kg feed	[52]
			1.2–2.0 mg/kg	[53]
	In vitro	human epithelial cells	0, 25, 500, 1000 bg/mL	[54]
		Cac0-2	50, 500, 5000 ng/mL	[55]
			250–1000 ng/mL	[56]
FB1	In vivo	pigs	6 mg/kg	[24]
ZEA	In vivo	pigs	1, 2, 3 mg/kg	[57]

**Table 5:** Toxicity studies: The toxic effects of mycotoxins on the intestinal mucosal immune barrier.

Mycotoxin	Species	Toxicity	References
AFB1	broilers	(1) Reduce the humoral immune function of the ileal mucosa in broilers; (2) Reduce the mRNA expression levels of immunoglobulins, such as IgA, pIgR, IgM, and IgG.	[50,58]
DON/FB1	pigs	Reduce the IgG content in serum and lymphocyte proliferation in piglets	[24]
ZEA	pigs	(1) Increase the levels of IgG and IgM in serum; (2) Decrease proportion of neutrophils indicates.	[57]
DON	human epithelial intestine 407 cells	Increase the level of IL-8 in human intestinal epithelial cells	[54]
	Caco-2	(1) A dose-dependent increase in IL-8 was shown in Caco-2 cells; (2) Increase the expression of IL-1-α, IL-1β, and TNF-α.	[55,56,59,60]
	pigs	(1) DON do not alter the expression of TGF-β, IFN-γ, IL-4, and IL-6 mRNA in the pig ileum following a short, low-dose regimen; (2) Reduce the expression of IL-1β and IL-8 in the pig ileum following a longer-term, low dose regimen. (3) Short-term, high-dose exposure to DON significantly upregulate TNF-α, IL-1β, IFN-γ, IL-6, and IL-10 levels in the ileum and jejunum of pigs	[24,52,53]
	mouse	(1) Induce the recruitment of regulatory B cells; (2) Activation of regulatory T cells and dendritic cells in the mesenteric lymph nodes.	[51]

**Table 6:** In vivo studies on the effects of different mycotoxins on intestinal mucosal biological barriers in different species.

Mycotoxins	Species	Dose	Reference
AFB1	rats	5, 25, 75 μg/kg BW	[64]
OTA	rats	0, 70, 210 μg/kg BW	[65]
ZEA	pigs	40 μg/kg BW	[66]
DON	pigs	12 μg/kg BW	[66]
	broilers	0, 2.5, 5, 10 mg/kg feed	[67]
	rats	100 μg/kg BW	[68]
FBs	pigs	0.41 mg/kg FB1 + 0.17 mg/kg FB2	[69]

BW = Body Weight.
